# Reducing Potentially Excess Deaths from the Five Leading Causes of Death in the Rural United States

**DOI:** 10.15585/mmwr.ss6602a1

**Published:** 2017-01-13

**Authors:** Macarena C. Garcia, Mark Faul, Greta Massetti, Cheryll C. Thomas, Yuling Hong, Ursula E. Bauer, Michael F. Iademarco

**Affiliations:** 1 Center for Surveillance, Epidemiology, and Laboratory Services, CDC; 2 National Center for Injury Prevention and Control, CDC; 3 National Center for Chronic Disease Prevention and Health Promotion, CDC

## Background

**FIGURE 1:**
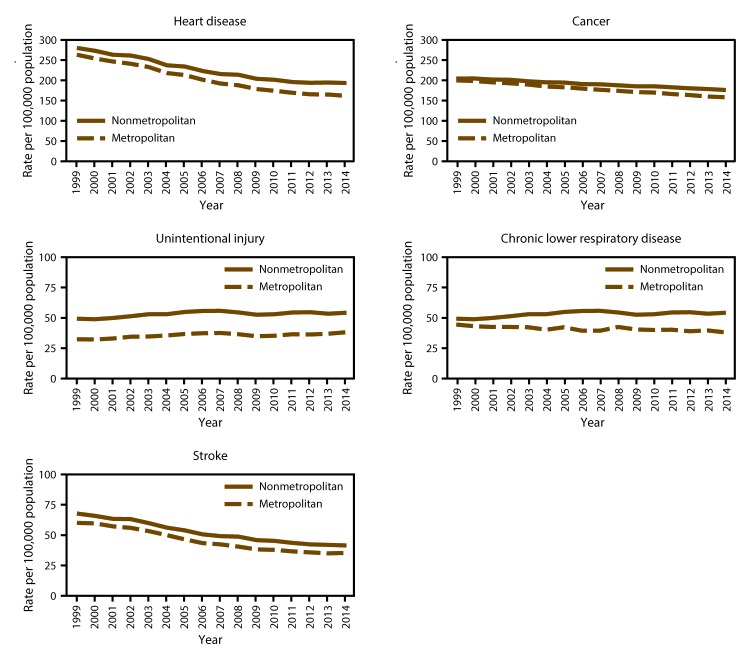
Age-adjusted death rates among persons of all ages for five leading causes of death in nonmetropolitan and metropolitan areas,* by year — National Vital Statistics System, United States, 1999–2014 **Source: **Moy E, García MG, Bastian B, et al. Leading causes of death in nonmetropolitan and metropolitan areas—United States, 1999–2014. MMWR Surveill Summ 2017;66(No. SS-1). * Nonmetropolitan and metropolitan areas were identified using the Office of Management and Budget’s 2013 county-based classification scheme. (**Source: **Office of Management and Budget, White House. Revised delineations of metropolitan statistical areas, micropolitan statistical areas, and combined statistical areas, and guidance on uses of the delineations of these areas. Washington, DC: Office of Management and Budget; 2013. https://www.whitehouse.gov/sites/default/files/omb/bulletins/2013/b13-01.pdf)

**FIGURE 2:**
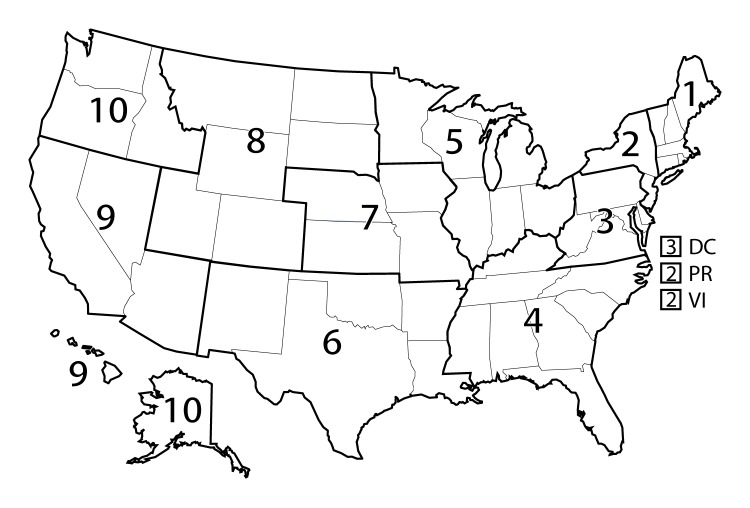
U.S. Department of Health and Human Services public health regions* **Source: **Moy E, García MG, Bastian B, et al. Leading causes of death in nonmetropolitan and metropolitan areas—United States, 1999–2014. MMWR Surveill Summ 2017;66(No. SS-1). * *1 *= Connecticut, Maine, Massachusetts, New Hampshire, Rhode Island, and Vermont; *2 *= New Jersey, New York, Puerto Rico, and the U.S. Virgin Islands (Mortality data for residents of U.S. territories were excluded.); *3 *= Delaware, District of Columbia, Maryland, Pennsylvania, Virginia, and West Virginia; *4 *= Alabama, Florida, Georgia, Kentucky, Mississippi, North Carolina, South Carolina, and Tennessee; *5 *= Illinois, Indiana, Michigan, Minnesota, Ohio, and Wisconsin; *6 *= Arkansas, Louisiana, New Mexico, Oklahoma, and Texas; *7 *= Iowa, Kansas, Missouri, and Nebraska; *8 *= Colorado, Montana, North Dakota, South Dakota, Utah, and Wyoming; *9 *= Arizona, California, Hawaii, and Nevada; *10 *= Alaska, Idaho, Oregon, and Washington.

**FIGURE 3:**
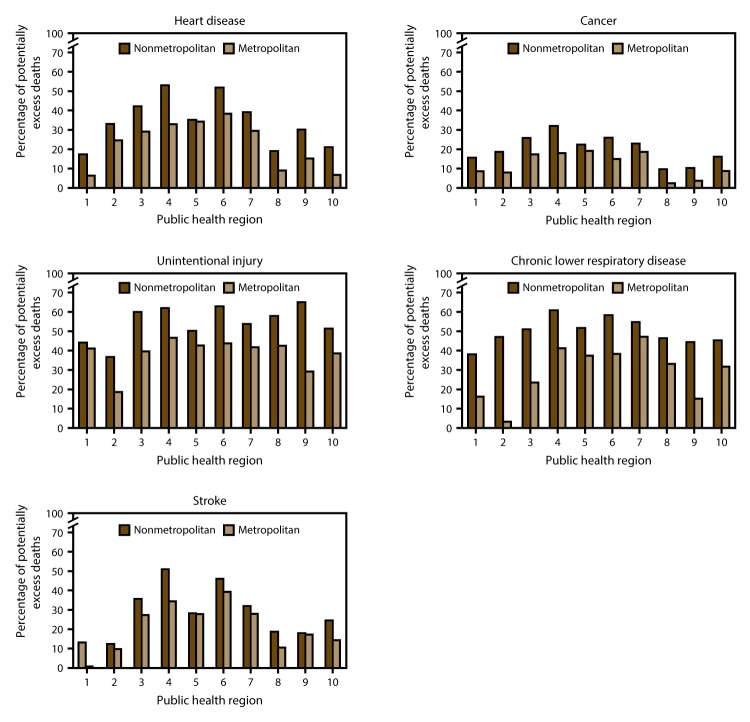
Percentage of potentially excess deaths* among persons aged <80 years for five leading causes of death in nonmetropolitan and metropolitan areas,† by year and public health region§ — National Vital Statistics System, United States, 2014 **Source: **Moy E, García MG, Bastian B, et al. Leading causes of death in nonmetropolitan and metropolitan areas—United States, 1999–2014. MMWR Surveill Summ 2017;66(No. SS-1). * For each age group and cause, the death rates of the three states with the lowest rates during 2008−2010 (benchmark states) were averaged to produce benchmark rates. Potentially excess deaths were defined as deaths among persons aged <80 years in excess of the number that would be expected if the age group−specific death rates of the benchmark states occurred across all states. † Nonmetropolitan and metropolitan areas were identified using the Office of Management and Budget’s 2013 county-based classification scheme. (**Source: **Office of Management and Budget, White House. Revised delineations of metropolitan statistical areas, micropolitan statistical areas, and combined statistical areas, and guidance on uses of the delineations of these areas. Washington, DC: Office of Management and Budget; 2013. https://www.whitehouse.gov/sites/default/files/omb/bulletins/2013/b13-01.pdf). § *1 *= Connecticut, Maine, Massachusetts, New Hampshire, Rhode Island, and Vermont; *2 *= New Jersey and New York; *3 *= Delaware, District of Columbia, Maryland, Pennsylvania, Virginia, and West Virginia; *4 *= Alabama, Florida, Georgia, Kentucky, Mississippi, North Carolina, South Carolina, and Tennessee; *5 *= Illinois, Indiana, Michigan, Minnesota, Ohio, and Wisconsin; *6 *= Arkansas, Louisiana, New Mexico, Oklahoma, and Texas; *7 *= Iowa, Kansas, Missouri, and Nebraska; *8 *= Colorado, Montana, North Dakota, South Dakota, Utah, and Wyoming; *9 *= Arizona, California, Hawaii, and Nevada; *10 *= Alaska, Idaho, Oregon, and Washington.

In 2014, the all-cause age-adjusted death rate in the United States reached a historic low of 724.6 per 100,000 population (*1*). However, mortality in rural (nonmetropolitan) areas of the United States has decreased at a much slower pace, resulting in a widening gap between rural mortality rates (830.5) and urban mortality rates (704.3) (*1*). During 1999–2014, annual age-adjusted death rates for the five leading causes of death in the United States (heart disease, cancer, unintentional injury, chronic lower respiratory disease (CLRD), and stroke) were higher in rural areas than in urban (metropolitan) areas (Figure 1). In most public health regions (Figure 2), the proportion of deaths among persons aged <80 years (U.S. average life expectancy) (*2*) from the five leading causes that were potentially excess deaths was higher in rural areas compared with urban areas (Figure 3). Several factors probably influence the rural-urban gap in potentially excess deaths from the five leading causes, many of which are associated with sociodemographic differences between rural and urban areas. Residents of rural areas in the United States tend to be older, poorer, and sicker than their urban counterparts (*3*). A higher proportion of the rural U.S. population reports limited physical activity because of chronic conditions than urban populations (*4*). Moreover, social circumstances and behaviors have an impact on mortality and potentially contribute to approximately half of the determining causes of potentially excess deaths (*5*).

Potentially excess deaths (also described as potentially preventable deaths) are defined as deaths among persons aged <80 years in excess of the number that would be expected if the death rates for each cause were equivalent across all states to those that occurred among the three states with the lowest rates (*6*,*7*). Although not all potentially excess deaths can be prevented, many might represent deaths that could be prevented through improved public health programs that support healthier behaviors and neighborhoods and better access to health care services.

## Reducing Potentially Excess Deaths in Rural Areas of the United States

In 2014, approximately 62% of all 1,622,304 deaths in the United States were related to the five leading causes of death (6). During 2014, the number of potentially excess deaths from the five leading causes in rural areas was higher than those in urban areas (*8*). Targeted, needs-based prevention efforts, combined with improved access to treatment for chronic conditions, might reduce the rural-urban gap in age-adjusted death rates and potentially excess deaths from the five leading causes of death.

## Heart Disease, Stroke, and Chronic Lower Respiratory Disease

Heart disease, stroke, and CLRD share several substantial co-morbidities from individual behavioral and social risk factors (*9*–*12*). The percentage of potentially excess deaths from these three causes is higher in rural than urban areas in all 10 regions (*8*). In addition, potentially excess deaths among regions vary substantially. For example, the percentages of excess deaths in urban areas in regions 3, 4, 5, 6 and 7 related to stroke are higher than even the rural area percentages in regions 1, 2, 8, 9, and 10. For these three causes of potentially excess deaths, the highest urban percent in one or more regions is higher than the lowest rural percentages in three or more regions. Stroke is the most prominent example: urban excess deaths in regions 4 (South East) and 6 (New Mexico, Texas, Oklahoma, Arkansas, and Louisiana) far outpace rural excess deaths in regions 1, 2, 5, 7, 8, 9 and 10. In region 5 (Great Lakes), rural-urban differences in potentially excess deaths nearly disappeared for heart disease and stroke. Additional research is needed to understand the causes of these differences and what can be learned from both the broad regional differences across the United States and the near elimination of rural-urban differences in the Great Lakes region.

Tobacco use increases the risk for developing and dying from heart disease, stroke, and CLRD. Cigarette smoking is the leading cause of preventable disease and death in the United States (*13*) and is the most substantive risk factor for CLRD (*10*). Nationally, the prevalence of cigarette smoking among adults living in rural counties is higher than in urban counties, and smoking rates differ markedly by region, making tobacco use a likely leading cause of differential mortality between urban and rural areas (*4*). Understanding where tobacco use in rural areas is higher than urban areas can help prioritize resources to reduce tobacco use and secondhand smoke exposure and begin to address the increasing numbers of CLRD-related deaths in rural areas.

Heart disease and stroke mortality rates are decreasing in both rural and urban areas. However, this improvement in mortality trends is plateauing and the rate of decline for heart disease in rural areas is slowing relative to urban areas, thus increasing the differential rural-urban gap in mortality rates (*8*). In 22 states, including rural states such as Alaska, Idaho, Kentucky, Montana, Vermont and West Virginia, the number of deaths attributed to heart disease declined below the number of cancer deaths for the first time (*8*). For stroke, the difference in the number of deaths in rural and urban areas remained constant, with substantive declines in both rural and urban areas (*8*). In 2013, stroke declined from the third to the fifth leading cause of death in nonmetropolitan areas (*14*). Lack of physical activity, poor nutrition (especially diets high in calories, sodium, added sugars, and saturated fat), and associated obesity are major risk factors for hypertension and diabetes (*15*). These risk factors and conditions contribute substantially to heart disease and stroke death rates in the United States and are more prevalent in rural areas than urban areas. Obesity has been linked to a variety of serious chronic illnesses, including diabetes, heart disease, cancer, and arthritis (*16*–*18*). From 1960 to 2010, the proportion of adults in the United States who were overweight or obese increased from 40.5% to 66.1% (*19*). Self-reported obesity was higher in rural areas than urban areas and increased with increasing levels of rurality (*4*). Regular physical activity and improved physical fitness offer numerous health benefits, including reduced risk for cardiovascular disease, diabetes, obesity, some cancers, and musculoskeletal conditions (*20*). Despite evidence of modest increases in the prevalence of physical activity and improved nutrition, the prevalence rates of hypertension and diabetes have not been improving over time (*21*,*22*). However, a greater percentage of adults with hypertension are controlling their blood pressure (*23*), and since 2006 there has been a sustained decline in the incidence of diabetes and a plateauing in the prevalence of diabetes nationally (*24*).

Barriers to health care access result in unmet health care needs that include, but are not limited to, a lack of preventive and screening services, treatment of illnesses (*25*) and timely urgent and emergency services (*26*). Residents of rural areas experience many of these barriers. Specifically, rural counties in the United States have a higher uninsured rate (*27*); experience health care workforce shortages (approximately only 11 percent of all physicians choose to practice in rural settings) (*28*); often lack subspecialty care (e.g., oncology), critical care units, or emergency facilities (*29*); have limited transportation options; and experience longer time to services caused by distance (26). Differential access to quality health care (*25*), including timely access, likely contributes to rural-urban gaps in mortality rates and potentially excess deaths. For example, persons with CLRD and unmet health care needs in rural areas can experience serious life-threatening respiratory episodes, and the lack of timely access to emergency care could affect survival. In contrast, the parallel mortality trends for stroke might be explained by the success of complementary programs that improve the quality of stroke care. These programs (CDC’s *Paul Coverdell National Acute Stroke Program*, the American Heart Association/American Stroke Association’s *Get with the Guidelines* program, and The Joint Commission’s Certification for Primary Stroke Centers program) organize systems that coordinate acute stroke care across both urban and rural hospitals (*30*). However, comparable programs to improve cardiac care have not been implemented in rural areas, which might account for divergent trend in myocardial infarction mortality rates.

## Cancer

During 2003–2012, the overall cancer-related age-adjusted death rate decreased by 1.5% per year (*31*); however, rates declined less in rural than urban areas (*8*). Age-adjusted death rates from cancer have mirrored decreases in the prevalence of risk factors such as tobacco use, which is a shared risk factor with heart disease, stroke, and CLRD (*10*–*12*), and increases in cancer screening, vaccinations, and improvements in treatment (*31*). Differences in these death rates might reflect higher prevalence of tobacco-use and obesity in rural areas (*4*) and lack of access to cancer screening services, follow-up to abnormal tests, quality care for cancer patients, and cancer survival care (*32*).

To address the rural-urban gap in cancer-related potentially excess deaths, comprehensive approaches that encompass the cancer continuum (e.g., prevention, early detection, treatment, and survivorship) are needed at the local and state level to reduce risks associated with potentially excess deaths from cancer in rural areas (*33*). Comprehensive cancer-control programs are funded by CDC in 50 states, the District of Columbia, seven tribes and tribal organizations, and seven U.S. territories and Pacific Island jurisdictions (https://www.cdc.gov/cancer/ncccp/about.htm). These programs build coalitions that develop and implement strategic plans for cancer prevention and reduce morbidity and mortality for persons affected by cancer.

CDC also supports cancer screening programs that address health disparities among adults who are uninsured or underinsured (*34*,*35*), which is a common characteristic among rural populations (*32*). Historically, funding for the National Breast and Cervical Cancer Early Detection Program and the Colorectal Cancer Control Program has focused on direct screening services. Since 2012, emphasis has shifted to population-based approaches, such as partnering with health systems to implement evidence-based interventions to increase population-level screening (*36*), including provider reminders for persons who are due for cancer screening (*37*).

As differences in cancer-related death rates are addressed at the local and state level, opportunities are available to address them at the federal level. The Cancer Moonshot is focused on accelerating the understanding of cancer and its prevention, early detection, treatment, and cure, including improving access and care (*38*). Although some components of this federal initiative target the genomic level, when combined with population-based approaches, the rural-urban gap in cancer-related deaths rates might be reduced.

## Unintentional Injury

During 2008–2010, the annual age-adjusted death rates for unintentional injury were highest in rural counties (*8*). During 1999–2014, the age-adjusted death rates for unintentional injuries were approximately 50% higher in rural areas than urban areas (Figure 1). Several factors explain the wide gap in rural-urban death rates from unintentional injuries. First, unintentional injury burden is higher in rural areas because of severe trauma associated with high speed motor vehicle traffic-related deaths (*4*). Second, rates of opioid analgesic misuse and overdose death are highest among poor and rural populations (*39*). Third, behavioral factors (e.g., alcohol impaired driving, seatbelt use, and opioid prescribing) contribute to higher injury rates in rural areas (*40*–*42*). Fourth, access to treatment for trauma and drug poisoning is often delayed when the injury occurs in rural areas. For life-threatening injury, higher survival is associated with rapid emergency treatment (*43*,*44*). Because of the geographic distance involved, emergency medical service (EMS) providers who operate ambulances take longer to reach injured or poisoned patients in rural areas. Moreover, ambulatory transport to the optimal treatment facility also can take longer because of increased distance to the treatment facility. Most life threatening trauma is best treated in advanced trauma centers, which are usually located in urban areas; care at these centers has been associated with 25% lower mortality (*45*). Trauma centers have advanced equipment and specialized staff available 24 hours, 7 days a week. Such care also includes access to advanced neurosurgical care, which is important because approximately one third of all injury-related deaths involve a traumatic brain injury. Regulatory restrictions and EMS capability and certification to treat drug overdose cases with naloxone at the scene of overdose events also might be a factor in higher opioid poisoning-related deaths in rural areas.

Interventions to address the disproportionate unintentional injury death rates in rural areas include increased adherence to guidelines for triaging ambulatory transport destinations (*46*), changing state rules to expand the types of EMS providers that can administer naloxone to reverse a drug overdose (*47*), and enforcement of motor vehicle seatbelt and alcohol laws to reduce motor vehicle crashes (*48*). Educating rural opioid prescribers on the opioid guideline (*49*) and better access to opioid agonist medication-assisted treatment programs probably would benefit rural communities with high opioid use disorder rates (*50*). Quicker access to definitive trauma and opioid dependency treatment and additional interventions are needed to reduce unintentional injury deaths in rural areas.

## Conclusion

In the United States, there is a rural-urban gap in age-adjusted death rates and potentially excess deaths from the five leading causes of death. Rural communities experience higher age-adjusted death rates and a higher number of potentially excess deaths from the five leading causes compared with urban areas. Higher death rates and potentially excess deaths are often associated with various interconnected societal, geographic, behavioral, and structural factors. Historic trends indicate that focusing on access to health care in rural areas of the United States alone is not sufficient to adequately address complex health outcomes, including mortality among rural populations (*3*). Consistent with the recommendations and best practices described in this report, approaches to address the nonuniform achievements in rural areas must focus on strengthening the health care delivery system while improving and increasing the integration of primary, specialty, and substance abuse services (*3*). Identifying structural and societal modifiable factors contributing to the gap between rural and urban mortality outcomes from the five leading causes of death is challenging. Additional analysis can yield results that inform the strategic alignment of resources with condition-specific needs. To reverse the widening gap in age-adjusted death rates from unintentional injuries between rural and urban areas, special attention should be given to designing, implementing, and monitoring locally informed initiatives in rural communities for the effective prevention and treatment of opioid misuse, including treatment of opioid overdose. Needs-based allocation of resources can substantially impact rural health. Although rural communities are at higher risk for death from the five leading causes of death, funding to address risk factors is allocated on a population basis (*3*), often resulting in underfunded rural programs. An increased emphasis on need and epidemiologic burden of disease as major factors in targeting future allocation of public health and prevention funding might contribute, among other factors, to bridging the mortality gap from the five leading causes of death between rural and urban areas in the United States.
